# Editorial: How Fear and Stress Shape the Mind

**DOI:** 10.3389/fnbeh.2016.00024

**Published:** 2016-03-08

**Authors:** Luke R. Johnson

**Affiliations:** ^1^School of Psychology and Counselling, Translational Research Institute, Institute of Health and Biomedical Innervation, Queensland University of TechnologyBrisbane, QLD, Australia; ^2^Department of Psychiatry, Center for the Study of Traumatic Stress, Uniformed Services University School of MedicineBethesda, MD, USA

**Keywords:** amygdala, resilience, PTSD, anxiety, microanatomy, topography, ethology, context

How do fear and stress systems interact and how do they shape ongoing and future behavioral responses? In a classical definition of fear and stress, we think of threatening stimuli activating a species-specific defensive threat reaction. This defensive reaction triggers physiological stress responses including adrenal hormone release (for review see LeDoux, 2003, 2012; Johnson et al., 2012). Knowledge of the microanatomy of conditioned threat memory is developing however, knowledge of its interaction with stress mediated adrenal steroid systems is still emerging (LeDoux, 2003, 2012; Johnson and LeDoux, 2004; Prager and Johnson, 2009; Prager et al., 2010; Bergstrom et al., 2011, 2013a,b; Bergstrom and Johnson, 2014; Krugers et al.). Studies have identified the key role of the lateral amygdala and within this nucleus the microanatomy of Pavlovian fear/threat memory consolidation, reconsolidation, and extinction has begun to be revealed (Bergstrom et al., 2011, 2013a,b; Bergstrom and Johnson, 2014). This Frontiers Research Topic builds on previous research by addressing key questions that reveal unique aspects and mechanisms of how fear and stress shape the mind.

The fear neural circuitry includes; amygdala output circuits that directly activate the sympathetic nervous system and also the hypothalamic pituitary adrenal (HPA) axis, thereby including stress hormones in the negative emotional response (Radley). It is generally accepted that negative emotion involves a stress response, however what stress is and how it manifests in the body has been, and continues to be, vigorously investigated and debated. Radley summarizes detailed circuit tracing and connectivity approaches to understand the interaction between stress and fear systems in the brain. Proposing that the anterior bed nuclei of the stria terminalis (aBST) is the central point for regulation of chronic stress induced hyperactivity of the HPA axis. This GABA projecting nucleus, upstream of the PVH, receives convergent input from amygdala, prelimbic cortex, and other fear related nuclei. Aspects of amygdala anatomy and its control of HPA responding may underlie differences in mental responding to fear and stress (Johnson and LeDoux, 2004; Johnson et al., 2012; McGuire et al., 2013).

Krugers et al. describe a series of studies in animals and humans that highlight the key time course and mechanisms of stress hormones norepinephrine and glucocorticoids in facilitating fear memories. They describe short-term rapid activation of NE Beta and Mineralocorticoid receptors (MR) in the postsynaptic space leads to rapid insertion of AMPA receptors in the postsynaptic membrane. Over a longer period (hours), Glucocorticoid receptors (GR) acting through genomic mechanisms also drive insertion of AMPA receptors into the postsynaptic membrane. These authors found that these multiple complementary cellular mechanisms facilitate and strengthen memories of stressful events.

By identifying the fundamental mechanisms underlying structural changes in the fear system in response to threatening stimulus associations, Lamprecht describes changes to the actin cytoskeleton and suggests, that it may be essential for pre- and post- synaptic changes that occur in the dendrite spines (particularly in lateral amygdala and hippocampus) following fear conditioning. It was found that inhibitors of the actin cytoskeleton modify neuron structure and dampen long-term memory (Lamprecht).

Starting from the assumption that age is a risk factor for anxiety disorders (Pardon and Rattray, 2008; Shoji and Mizoguchi, 2011), Beracochea et al. used stressed middle-aged and non-stressed young adult mice to understand the interaction between the fear circuitry and its link with anxiety disorder, memory, and pharmacology. When administered benzodiazepines in specific dose range, stressed middle-aged mice became like young adult non-stressed mice, on a hippocampal memory task. This provides the first evidence of a dynamic interaction between benzodiazepines and corticosterone levels, indicating a reduced stress effect and improved memory performance.

Potential overlapping pathways between fear, stress, suicide, anxiety, and aging are identified by Choi et al., who found kinase gene expression levels increased in the prefrontal cortex of suicide victims compared to controls. Postnatal disruption of (kinase) genes by environmental factors may increase later pathophysiology increasing the risk of suicide. In addition to Kinase genes, other regulators of stress may be important indicators and pharmacological regulators of the amygdala-prefrontal cortex stress axis. McGuire et al. report that Neuropeptide Y (NPY) plays a role in integrating stress and emotion in part through regulation of CRH, and, that a dysregulation of NPY may leave an individual more exposed to the negative aspects of subsequent stress.

Nolte et al. summarize important work on how attachment experiences during development influence the development of anxiety and HPA axis sensitivity. They propose, that stress sensitivity characteristics that an infant is born with could represent in utero adaptation of stress regulation style of the mother. Thus, anxiety in the mother can be transferred from mother to child through dysregulation of the HPA axis. A person's sensitivity to developing post-traumatic stress disorder (PTSD) may be influenced by their genetic, development and environmental experiences.

PTSD is associated with dysregulated fear and stress systems. In an elegant article by Jovanovic and Norrholm, fear inhibition models are suggested to be possible translational tools for studying fear reduction in animals and humans. Facilitation of fear extinction mechanisms both, behaviorally, and pharmacologically, may produce therapeutic modification to underlying neural circuitry. They identify that decreased ability to reduce fear is a risk factor for the development of PSTD. Reduction of fear is context and time dependent. Huff et al. developed a sophisticated virtual reality procedure for context and cued fear in humans. They identified a time dependency and memory consolidation of context fear develops quickly. In contrast, memory consolidation of differential cued fear (CS+/CS−), develops slowly. These finding have important implications for understanding anxiety and testing anxiety in humans.

In a fresh and novel perspective for PTSD research in wild animals Clinchy et al. propose, that we need to know how real animals deal with real stress. They investigate the “predator model of PTSD” in which exposure to odor of the predator leads to long lasting changes in the brain and body, including to CRH and corticosterone, and to dendrite morphology. Predator exposure to wild prey animals has been shown to lead to 40% less offspring production and it is linked to glucocorticoid elevation in the parents. Multi-generational stress has been demonstrated in snowshoe hares which may increase an adaptive predator response in future offspring. Clinchy et al. propose, that trans-generation stress responses may be personally maladaptive but evolutionarily adaptive. If stress is maladaptive why does it persist? It may be a struggle to live with but not necessarily maladaptive to survival, thus maladaptive stress responses may make sense.

Throughout human history, every generation has arguably faced an epidemic of fear and stress associated mental trauma which frequently manifests as PTSD (Ursano et al., 2010). This epidemic afflicts past, present and future generations. The 11 studies presented provide a fresh perspective into how fear and stress systems interact and how they may influence the development of emotional and pathological states. How bodily stress systems interact with the neurobiology of fear and mental health continues to be an important question in neuroscience (Prager et al.). Future studies will need to revisit and solve fundamental mechanisms of emotion in order to effectively understand and treat pathologies of fear, stress, and trauma.

## Author contributions

The author confirms being the sole contributor of this work and approved it for publication.

### Disclaimer

The views expressed in this article are those of the authors and do not necessarily reflect the official policy or position of the Department of Defense, nor the U.S. Government.

### Conflict of interest statement

The author declares that the research was conducted in the absence of any commercial or financial relationships that could be construed as a potential conflict of interest.
